# Social Work in America

**Published:** 1920-02-21

**Authors:** 


**THE PUBLIC WELL-BEING**—*(continued)*

### Social Work in America.

### More Enthusiasm and Easier Finance.

"An Englishwoman in American" is the title of a series of informative articles which have been appearing in the *Manchester Guardian* over the name of Muriel Harris, and there is one on social work which contains so much that is interesting and stimulating, that we reproduce the main points. It is by no means demonstrated by the article that we in England are generally behind the United States in our methods—in fact, it was Miss Lillian Wald, founder of the Henry Street Settlement, New York, who said of social work in America that, while it had higher peaks, the English average was perhaps higher. But it is always valuable and refreshing to see social work from some other nation's angle.

The American is usually out for peaks, and it is the peaks which first catch the eye, and, at the same time, help you to get your bearings. Social work of any kind inevitably leads to or is connected with nursing and public health. In this connection Red Cross nursing and settlement nursing have received a great impetus from the war, and the demand for nurses in every direction is very much greater than is the supply. With the great demand, scholarships, and new methods of training, new opportunities of experience in settlement work and a great new enthusiasm for the work are signs that, as far as nursing goes, some new "peaks" are being established in America. A great anti-tuberculosis campaign is a direct outcome of experience gained during the war of the ravages of this disease. The new census which is shortly to be taken is being conducted on a basis which will provide important new statistics in this direction. Many women of means are interesting themselves in the campaign, and public health, from being social, is also becoming fashionable. A good many girls, too, who acted during the war as the equivalent of our V.A.D.s for the first time had their eyes opened to the crying needs of the poorer classes. They now have an interest in all kinds of public health work which before the war would have been impossible. There is also the class—quite a large one here—to whom war-work of any kind proved absorbing when they had never had anything absorbing to do before. A great many of these women felt that they could not go back to a life devoid of the interest of the work. Thus women who have been plodding away steadily for years at, say, medical work among the poor and distressed now find themselves overwhelmed with volunteers for social work of some kind.

The Henry Street Nursing Service is one of the pioneer foundations for nursing in the home. How great is the need of it can be judged from the fact that only 10 per cent. of New York's sick go to the hospital. There is, indeed, considerably more prejudice against hospitals than is the case in England. This is partly due to the many and coherent nationalities in New York, all with different customs and conventions, and many in ignorance, and therefore suspicious, of the institutions of another country. In 1917, the Henry Street nurses—134 in number—made 277,170 visits to 32,753 patients. They attend all kinds of cases, nurse, dress, bandage, teach the family how to carry on the treatment. Chiefly, of course, their work deals with maternity cases, and there is a night service which can be called upon at any moment. The great value of this service is, of course, that connected with the Settlement, it is well known in other capacities to the people in whose quarter it functions. Probably the children have attended classes there; probably they belong to one of its many clubs. Perhaps the mothers know other mothers who belong to one of the clubs and like it. The Henry Street Settlement works hand in hand with the Red Cross, which is offering a number of nursing scholarships, Henry Street provides the practical experience and a most valuable training.

There is also a Department of Nursing and Health at Columbia University, and these semi-public institutions work in and out in a co-ordinating manner which to the outside eye is admirable. The women's City Club, including among its members some of the best-known names in New York—best known, that is to say, because of their achievements—has entered into the matter of maternity nursing and has established with Henry Street a maternity clearing-house, to which all organisations giving maternity service refer their cases. Infant welfare centres are also greatly on the increase. This is largely due to the war, the loss of life in battle, while proportionately small in America, yet serving to point the moral of conserving young life. Several centres have been established lately, and with each successive centre the need for more becomes increasingly apparent. It was in 1909 that the Metropolitan Life Insurance Company undertook the nursing of its policy holders. This Company now employs the Henry Street nurses to care for their patients. Factories and workshops are also learning slowly—on the lines of our own welfare work—that it pays to study the health of the workers, and there is considerable increase just now in the demand for nurses both by them and by trade unions, mutual benefit societies, and so forth. Very great efforts are being made to send nurses in larger numbers to the rural districts.

Social work includes the usual settlement work that is carried on in England, but it is carried on in a slightly different spirit. Henry Street cares for the young, gives instructions and entertainment, teaches children to play as well as to work, carries on in its beautiful Neighbourhood Playhouse a first-rate dramatic training for the young Russian Jews among whom it works. The difference of spirit generally lies perhaps partly in the financial situation. To the outsider the money problem seems less harassing. Everything is done on a lavish scale, which unquestionably oils the wheels of work. Works of this kind in America are apt to depend upon a few large subscribers, where in England they depend upon a number of people of moderate means. In America sufficient enthusiasm will raise money with comparative ease. In England more guarantees than enthusiasm are necessary. In America nobody minds experimenting. Chance is the genius of the country, and failures are quickly forgotten. Promoters, therefore, of social work do not give the harassed impression that in England is so often the case, and, with comparative freedom from financial worries, they are able to give themselves freely to the work in hand. Finally there is about this work more enthusiasm and more faith. You cannot help having faith in a thing so well and so normally run. Workers work hard, but in their leisure hours they have comfort and they have amusement. They are kept very much in touch with the outside world, and there is nothing segregational or vocational about them. It is because of its normality, its recognition of the ordinary as well as the extraordinary life, that social work in America gives the impression of success. For this reason, too, the impetus given to it by the war would seem to be no temporary affair, but an agent by means of which peaks may eventually be turned into plateaux.

